# Supplementary material for: Field evidence of Trypanosoma cruzi infection, diverse host use and invasion of human dwellings by the Chagas disease vector in Florida, USA
Source: PLoS Negl Trop Dis. 2025 Jul 7;19(7):e0012920. doi: 10.1371/journal.pntd.0012920 (PMC12233274; doi:10.1371/journal.pntd.0012920)
Supplement: Attachment — Submitted filename: Beatty 2025.pdf [file pntd.0012920.s002.pdf]

Introduction

L70: vector's **infected** excreta- indicate that the vector must be infected

Methods

L144: what is Larry's lighthouse- a vendor?

L145: specify 'structures'

L145: does 'walls around homes' refer to the external walls of homes?

Fig 1B: Give details of UV light trapping for both the light sheets and the light traps (give details separately for each trap type): UV wavelength, when they were set up (i.e., time of day), for how many hours the traps were employed each night, #locations at which light trapping was carried out, how many nights the traps were used. etc. Include what worked and what didn't, to the extent that other researchers can repeat (or not) the same methods.

L160: change 'received' to 'sent'

167: it's either 'received by' or 'sent to' but not 'received to'

L167: morphologically identified how? Using which key?

L183: were negative controls included in the DNA extractions? How did you control for human contamination?

L202 screened using which methods?

L202-203: fix grammar and repetitive 'from': '… utilizing similar methods from our group from previous studies among triatomines…'
L246-252: What e-value cutoff was used?

Results:

Table 4: insert subtotals for each taxon (eg amphibia, mammals, etc.)

General: How did they detect the wood roach DNA using vertebrate specific primers. Please provide all methodological details to the extent that it is repeatable.

416: fix grammar

417-418: how was the toad associated with *T. cruzi* detection beyond it being a host for a single blood meal from six different infected bugs? We know triatomines take many blood meals in a lifetime, so it is a stretch to label six infected bugs that fed on toads as an association with *T. cruzi* infection

Table 5: why did the authors choose to calculate CIs here but not in any of the other proportional the results? The CIs are not discussed anywhere in the text and are more of a distraction than informative since they are not given with any context or explanation.

Table 6: Break up last column into multiple columns, one for each type

Discussion

L524: change ‘defect’ to defecate
